# Emergency radiology: roadmap for radiology departments

**DOI:** 10.1007/s11604-025-01819-0

**Published:** 2025-06-20

**Authors:** Sonay Aydin, Bunyamin Ece, Vefa Cakmak, Burak Kocak, Mehmet Ruhi Onur

**Affiliations:** 1 Department of Radiology, Faculty of Medicine, Erzincan Binali Yildirim University, Erzincan, Türkiye; 2 Department of Radiology, Faculty of Medicine, Kastamonu University, Kastamonu, Türkiye; 3 Department of Radiology, Faculty of Medicine, Pamukkale University, Denizli, Türkiye; 4 Department of Radiology, Basaksehir Cam and Sakura City Hospital, Istanbul, Türkiye; 5 Department of Radiology, Faculty of Medicine, Hacettepe University, Ankara, Türkiye

**Keywords:** Emergency radiology, Mass casualty incidents, Teleradiology, Artificial intelligence, Operational workflow, Interdisciplinary collaboration

## Abstract

Emergency radiology has evolved into a significant subspecialty over the past 2 decades, facing unique challenges including escalating imaging volumes, increasing study complexity, and heightened expectations from clinicians and patients. This review provides a comprehensive overview of the key requirements for an effective emergency radiology unit. Emergency radiologists play a crucial role in real-time decision-making by providing continuous 24/7 support, requiring expertise across various organ systems and close collaboration with emergency physicians and specialists. Beyond image interpretation, emergency radiologists are responsible for organizing staff schedules, planning equipment, determining imaging protocols, and establishing standardized reporting systems. Operational considerations in emergency radiology departments include efficient scheduling models such as circadian-based scheduling, strategic equipment organization with primary imaging modalities positioned near emergency departments, and effective imaging management through structured ordering systems and standardized protocols. Preparedness for mass casualty incidents requires a well-organized workflow process map detailing steps from patient transfer to image acquisition and interpretation, with clear task allocation and imaging pathways. Collaboration between emergency radiologists and physicians is essential, with accurate communication facilitated through various channels and structured reporting templates. Artificial intelligence has emerged as a transformative tool in emergency radiology, offering potential benefits in both interpretative domains (detecting intracranial hemorrhage, pulmonary embolism, acute ischemic stroke) and non-interpretative applications (triage systems, protocol assistance, quality control). Despite implementation challenges including clinician skepticism, financial considerations, and ethical issues, AI can enhance diagnostic accuracy and workflow optimization. Teleradiology provides solutions for staff shortages, particularly during off-hours, with hybrid models allowing radiologists to work both on-site and remotely. This review aims to guide stakeholders in establishing and maintaining efficient emergency radiology services to improve patient outcomes.

## Introduction

Emergency radiology encompasses the imaging and care of critically ill or injured patients. As a formal subspecialty, emergency radiology is relatively recent; however, over the past 2 decades, it has developed into a significant branch of radiology [1, 2].

The primary obstacles in the emergency radiology practice area include the escalating imaging volumes and study complexity, along with heightened expectations from referring physicians and patients, all without a corresponding growth in the number of radiologists. Despite these challenges, several factors, including increased demand for staff and equipment, organization with 7/24 coverage for emergency imaging, advancements in imaging techniques, and growing body of scientific research, have substantially transformed the practice of emergency radiology [2–4].

The escalation in emergency imaging demands has been substantial. Emergency physicians request the prompt accessibility of all imaging modalities, superior quality imaging examinations, minimal waiting periods, immediate reporting, real-time post-processing (three-dimensional, perfusion imaging), and continuous service and/or coverage, 24 h a day, 7 days a week.

Because emergency imaging is increasingly important and more people visit the emergency room for imaging, emergency radiology services need to be better organized and provided by professionals. To provide the expected level of service, facilities must be properly designed and run, and emergency radiologists must be able to communicate clearly with clinicians. Modern artificial intelligence (AI) technologies and teleradiological resources can support radiologists in meeting these demands. The aim of this review is to provide an up-to-date and comprehensive overview of the key requirements of an effective emergency radiology unit.

### Role of emergency radiologist

Emergency radiologists play an active role in real-time decision-making in emergency services by providing continuous support 24/7. Comprehensive emergency imaging coverage can be achieved with radiologists who are preferably trained through an emergency radiology fellowship program. Emergency radiologists should be familiar with the imaging findings of a variety of disease processes that may be encountered in the emergency setting, covering different organ systems from head to toe for accurate imaging assessment [5]. Close communication and collaboration with emergency physicians, trauma surgeons, neurologists, and other specialists are essential for discussing, advising, and reporting imaging findings. This collaboration is crucial for providing fast and effective management of emergency imaging, especially in life-threatening conditions such as strokes, internal bleeding, fractures, pulmonary embolisms, and organ injuries. In addition to diagnostic tasks, emergency radiologists may also perform image-guided procedures, such as abscess drainage or central line placement [6].

**Fig. 1:**
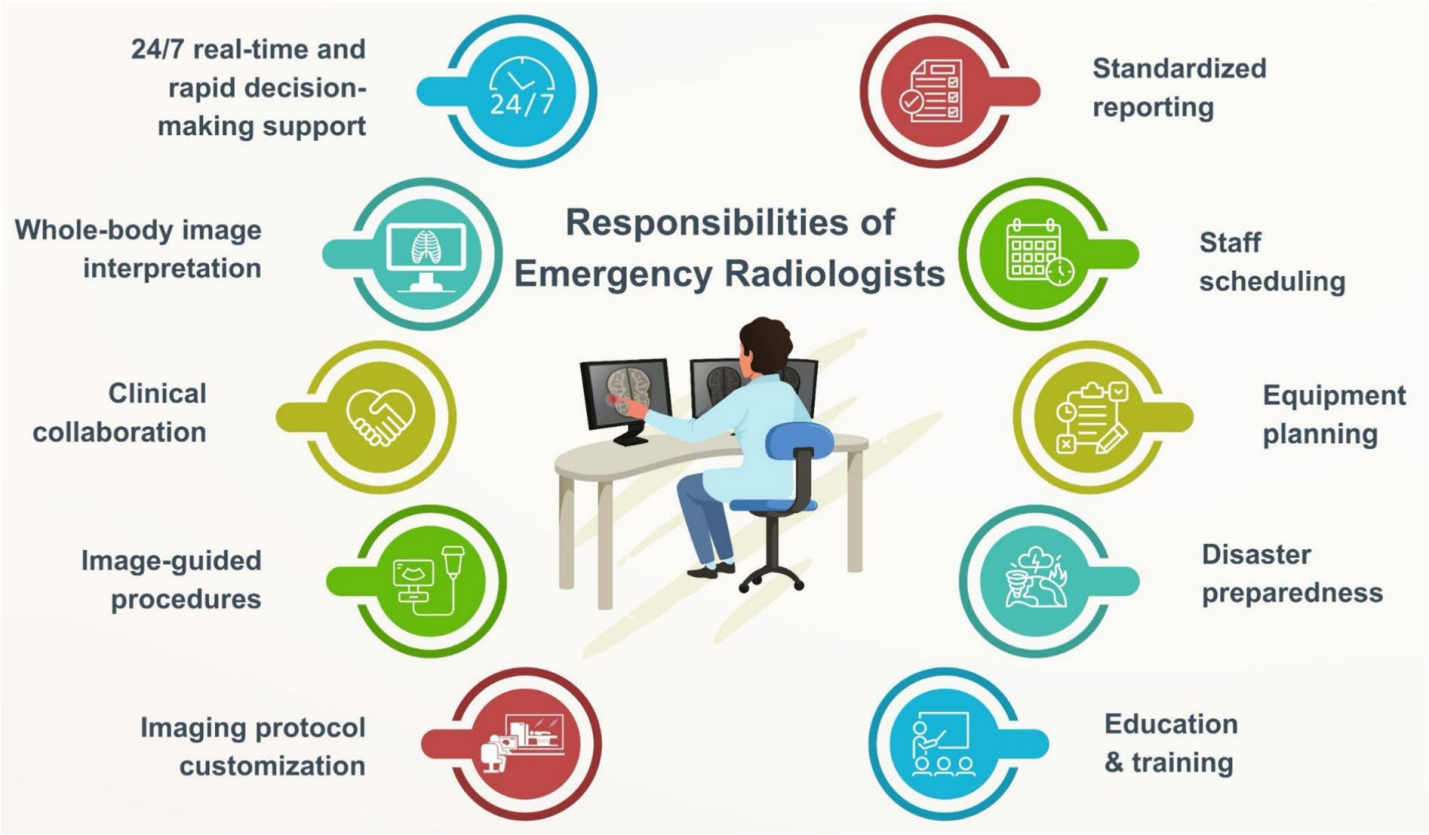
Multitasking responsibilities of emergency radiologists

Beyond interpreting images, emergency radiologists are responsible for organizing staff working hours, planning equipment, determining imaging protocols, and establishing standardized reporting styles. Imaging protocols should be tailored by emergency radiologists based on emergency indications and patient conditions (Fig. 1) [1]. Emergency radiologists serve as key figures in the preparedness of emergency radiology units for mass casualty incidents (MCIs), where a quick and appropriate response is needed for the overwhelming number of patients.

### Operational considerations in emergency radiology

In emergency radiology departments, the high volume of patients and the need for rapid diagnosis in various emergency pathologies, coupled with operational arrangements directly affecting patient outcomes, make operational considerations a critical process. From managing unpredictable workflows to ensuring fast imaging and reporting, every aspect of the emergency radiology unit must be carefully planned [7]. Operational considerations include essential elements such as personnel planning, equipment organization, and imaging management [7, 8].

1. Scheduling, staffing, and shift design

Efficient scheduling, appropriate staffing, and well-structured shift designs are essential for a smoothly functioning emergency radiology department. Given the dynamic and unpredictable nature of emergency imaging, a large proportion of the staff must be flexible, able to shift their focus and attention according to the tasks at hand, and capable of coping with an environment that can sometimes be chaotic, noisy, and stressful. In addition, ensuring sufficient productivity while preventing staff fatigue presents a significant challenge [8, 9]. To ensure continuous coverage, emergency radiology departments operate 24/7, requiring a robust shift-based work schedule (Fig. 1).

Common models suggested for emergency radiology scheduling include traditional shifts (day/evening/night), swing shifts to cover peak demand periods, and hybrid models that allow for flexible working hours. Among these arrangements, challenges such as increased fatigue during night shifts and decreased diagnostic accuracy may arise. Solutions such as circadian-based scheduling, power naps, or mandatory rest periods can be implemented to reduce these challenges [10].

In circadian-based scheduling, shifts are planned in a clockwise rotation (e.g., day → evening → night), utilizing a forward-facing shift rotation [9, 11]. Alternatively, shift rotations can be planned more regularly and uniformly (e.g., consecutive night shifts), keeping shift hours as consistent as possible, allowing the body to adjust to a new rhythm and avoid the negative effects of continuous changes [10].

Power naps typically involve short sleep breaks, usually lasting 10–20 min, taken during shifts or designated rest periods. Particularly before night shifts, a brief nap can improve performance throughout the shift. During a long night shift, especially at the circadian low point (e.g., between 2 and 4 AM), a short nap can help restore alertness. However, these naps must be kept short, as sleeping for more than 20 min can lead to deep sleep, which may result in sleep inertia [12–14].

Mandatory rest periods are breaks implemented to ensure that radiologists receive adequate rest during or between shifts. Mandatory rest periods can be scheduled within shifts, or adequate time can be provided between shifts to ensure proper sleep and recovery. In addition, the number of consecutive night shifts worked may be limited to prevent cumulative fatigue [15].

**Fig. 2:**
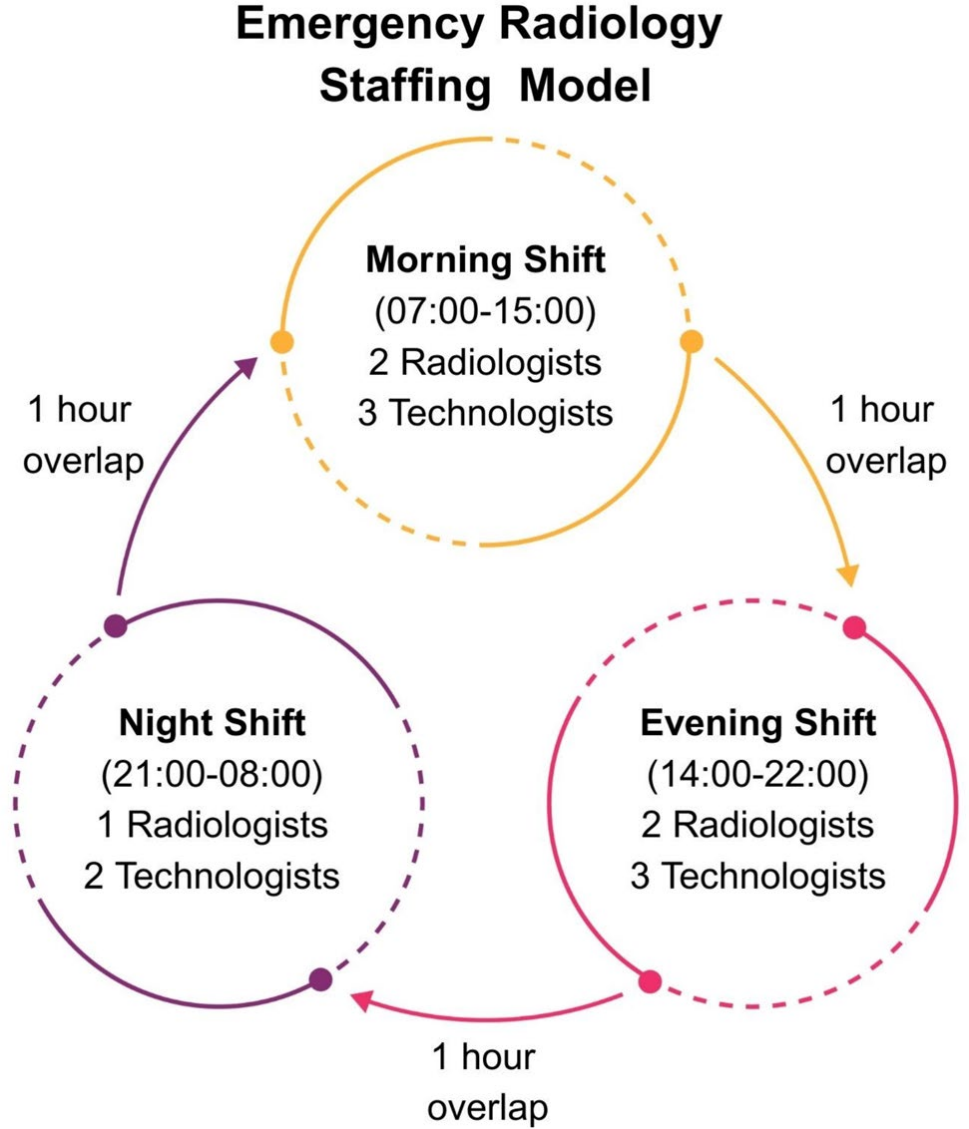
Emergency radiology staffing model

Optimizing staffing levels can be accomplished using historical data along with predictive analytical approaches to determine on which time or days patient volume increases. By modeling in this way, personnel needs can be more precisely determined. An example of a structured shift plan is illustrated in Fig. 2, which outlines the emergency radiology staffing model with defined overlaps to ensure continuity of care.

Through rotation programs, radiologists can be provided with regular practice in different modalities. In addition, mentorship programs can help transfer the knowledge of experienced specialists to younger radiologists. Regular skills assessments assist in identifying educational needs and maintaining quality standards. Structured training programs ensure that radiologists’ knowledge stays up to date. Moreover, simulation-based training and regular case review sessions encourage a culture of continuous learning and improve diagnostic accuracy [16].

2. Equipment organization

The physical layout and organization of imaging devices significantly impact workflow efficiency in emergency radiology. Primary imaging modalities such as X-ray and computed tomography (CT) should be strategically positioned close to the emergency department, taking patient flow into account [17]. Portable imaging solutions, such as mobile X-ray and point-of-care ultrasound (POCUS), are crucial for unstable or immobile patients. For some emergencies, quick access to magnetic resonance imaging (MRI) (e.g., for spinal injuries) and emergency interventional procedure rooms (e.g., for stroke thrombectomy) is necessary. Since routine MRI protocols often have long acquisition times, fast MRI protocols specifically designed for emergency radiology should be developed. The implementation of such fast MRI protocols requires collaboration between emergency radiologists and subspecialists with more expertise in MRI protocol design [18, 19].

Regular quality assurance, calibration, and preventive maintenance ensure consistency in image quality and minimize equipment downtime. In addition, having a backup system or a second scanner can help reduce workflow disruptions during equipment failure [20].

3. Imaging management: ordering, algorithm, protocols, reporting

**Fig. 3:**
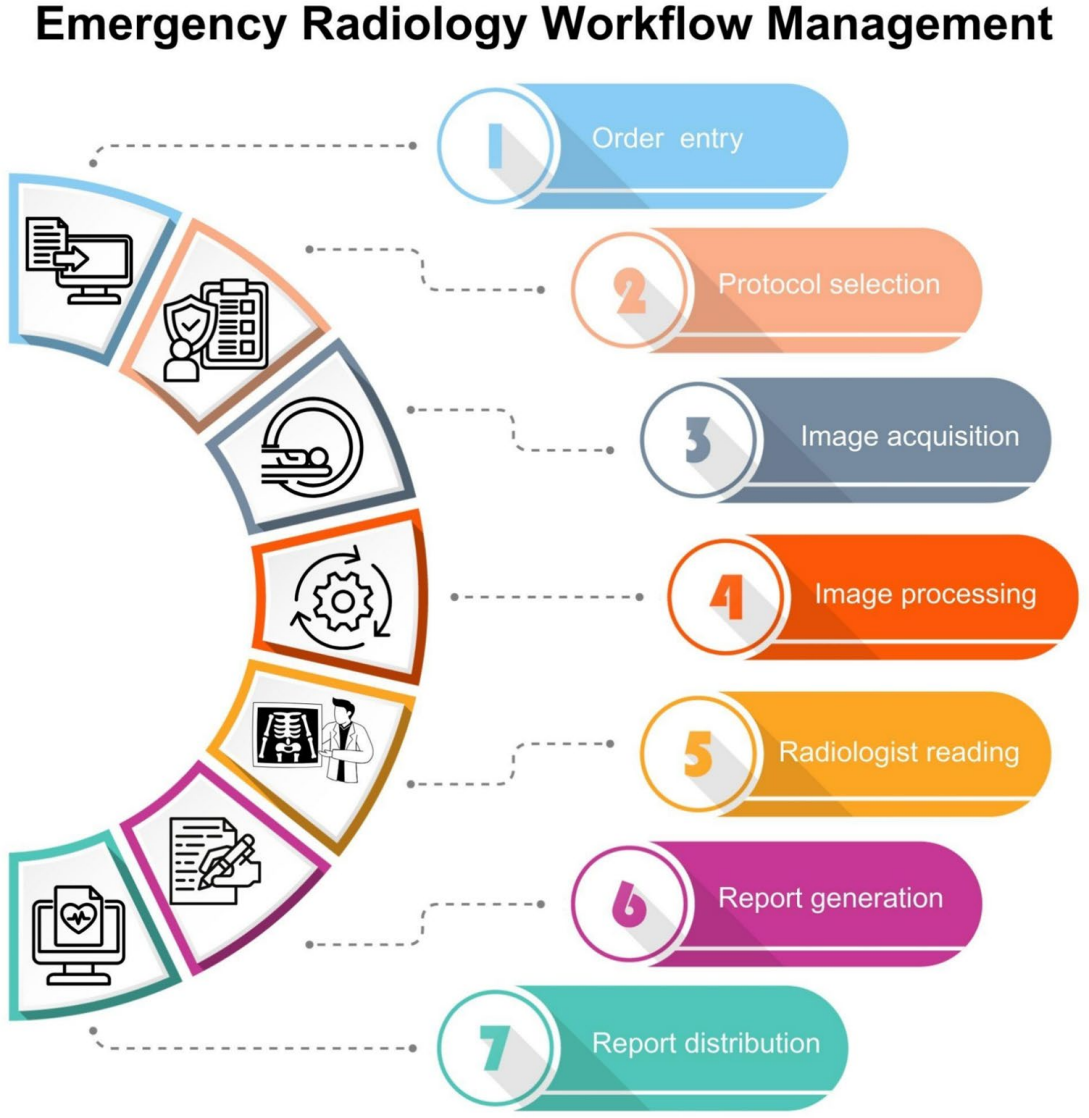
Emergency radiology workflow management. Standardized workflow ensures efficient emergency imaging management

Effective imaging management ensures that the right procedures are performed at the right time using the correct protocol. Structured ordering systems such as clinical decision support (CDS) systems, emergency severity index (ESI)-based prioritization, and the use of predefined ordering sets help prevent the overuse of imaging, optimize workflow, improve efficiency, and enhance patient care (Fig. 3).

Full-body imaging protocols for polytrauma patients [21], non-contrast CT, CT angiography, and CT perfusion imaging protocols for stroke patients, and low-dose imaging protocols for pediatric imaging should be standardized. These protocols reduce delays in imaging [22]. Minimizing delays is critical, as it has been reported that each additional 3 min spent in the emergency department increases the likelihood of death by approximately 1% [23].

The use of standardized templates for reporting workflow optimization enhances the clarity of reports through structured reporting and reduces reporting errors. The implementation of a closed-loop alert system ensures the immediate communication of life-threatening findings (e.g., aortic dissection, intracranial hemorrhage).

By creating a quiet reading station for the emergency radiologist near the emergency department, the radiologist can observe the imaging as it is being performed, when necessary, which may also help reduce routine over-scanning. In addition, the accessibility of the emergency radiologist can facilitate the establishment of social relationships with emergency department providers and improve communication [8].

Assessment of non-emergency pathologies (e.g., oncological assessment and staging, incidental lesions, etc.) in reports can be better performed by specialists with more knowledge and experience in these areas, who may also face less time pressure than those working in the emergency department. A system that allows non-emergency additional findings to be interpreted by the appropriate radiology specialist at a later time can improve efficiency and patient care in the emergency radiology department [8].

### Preparedness for mass casualty incidents

A mass casualty incident (MCI) is defined as any event in which the number of casualties impedes the standard operation of healthcare services and emergency response [24].

MCIs may arise from natural phenomena, such as tornadoes, floods, earthquakes, or pandemics, or they may be anthropogenic, whether accidental or deliberate, including transportation mishaps, industrial explosions, gas leaks, or acts of terrorism. The annual incidence of natural MCIs has increased fourfold over the past 30 years [25].

The correlation between the rising incidence of MCI and the utilization of imaging techniques underscores the critical role of radiology departments in MCI management and the necessity for appropriate preparations [24, 25]. MCI can lead to abrupt and complete interruption of radiology departments by interfering with electricity supply. An effectively devised emergency radiology department MCI plan and an organized emergency radiologist’s work list must encompass the assessment of all imaging modalities, including X-ray, ultrasound, CT, and MRI, and implement actions to restore any non-functional systems, if required in conjunction with radiologic technologists [25]. MCI preparedness plan should include battery-powered ultrasound and portable radiography-based secondary triage and redistribution of technologists, sonographers, and radiologists to these modalities [26]. Absence of radiology-focused pre-disaster power outage plan may result in inability to run a CT scanner on emergency power [27].

A well-organized radiology department should possess an MCI workflow process map detailing the sequence from patient transfer to image acquisition, processing, and interpretation. The departmental planning must encompass all aspects of labor, process, equipment, and execution. The rules should consider the inclusion of radiologists in direct hands-on care inside the emergency department, such as targeted assessment using sonography in trauma evaluations and image-guided interventions [28].

The initial stage in radiological assessment is the accurate identification of the patient, a process complicated by a significant influx of patients, some of whom may be incapable of self-identifying due to neurological impairments, hemodynamic instability, or psychological trauma [25].

**Fig. 4:**
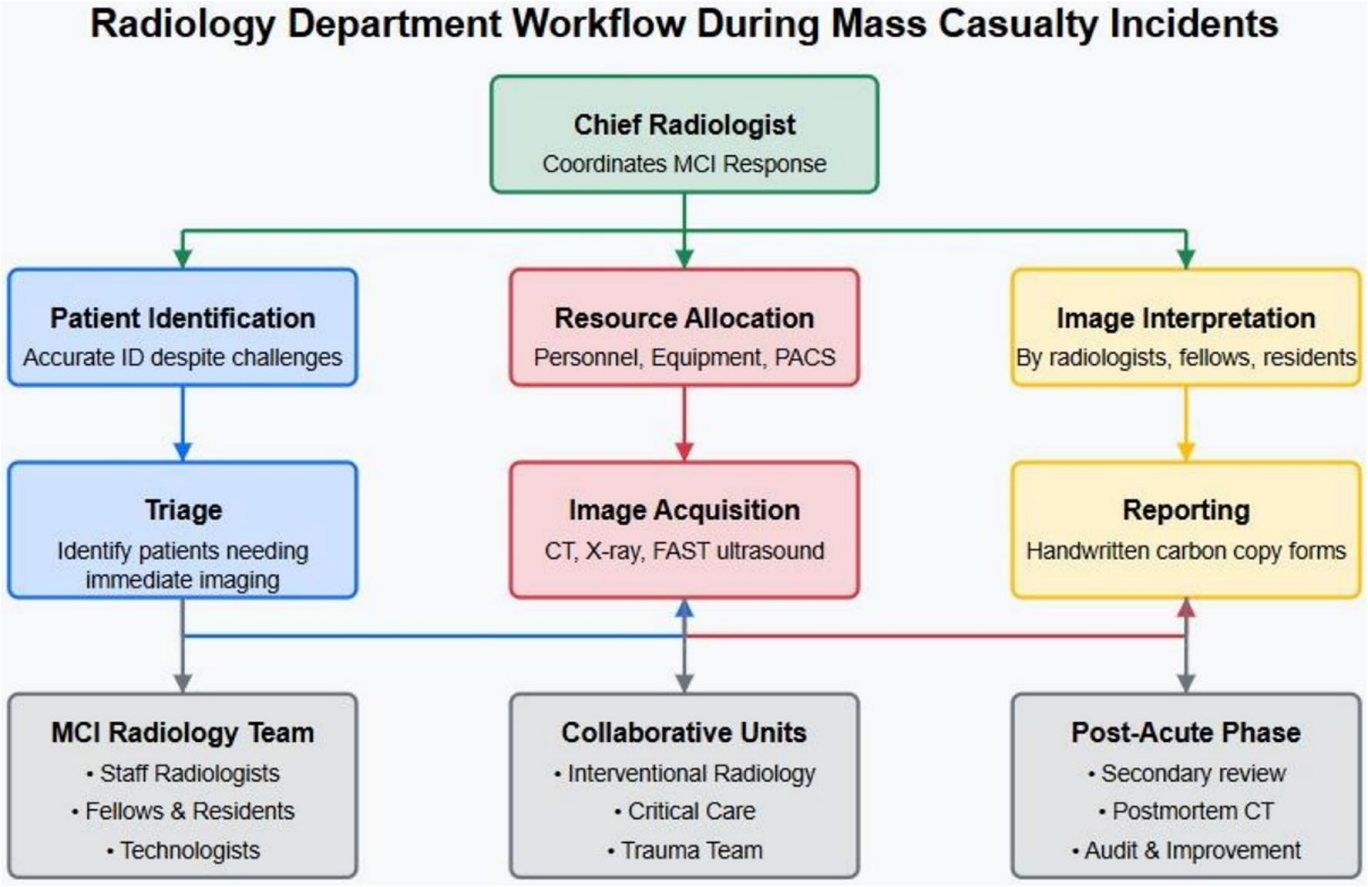
Workflow during mass casualty incidents

The radiology MCI team comprises staff radiologists, fellows, residents, and technologists who should be summoned to obtain and interpret findings. The chief radiologist must allocate responsibilities and tasks to the team members. The lead radiologist is expected to organize and implement the identification of patients needing immediate imaging (i.e., triage) and the necessary adjustments to ordinary institutional practices to accommodate an MCI. The quantity of scanners and reporting workstations to be implemented, together with the workflow structure, must be meticulously prearranged [24, 25, 28]. A sample radiology workflow model for MCIs, including task allocation and imaging pathways, is illustrated in Fig. 4.

If the digital communication systems are disrupted during an MCI, handwritten early reports on preprinted carbon copy tick box forms that record significant life-threatening injuries across each bodily system represent an efficient and effective method for conveying findings during an MCI. The significance of acutely conveying crucial non-traumatic findings is constrained; a further review in the days following the occurrence can serve as a safeguard.

Following the management of the acute episode of an MCI, postmortem computed tomography, commonly known as virtual autopsy, provides a pragmatic, economical, and insightful alternative to conventional autopsy [24, 25].

### Collaboration with emergency physicians

Best practices in emergency radiology necessitate precise and efficient communication with emergency physicians, including surgeons, neurologists, and other emergency care clinicians. This collaboration yields various benefits, including accurate assessment of patients with a time-oriented diagnostic and therapeutic approach to improve patient outcomes, a multidisciplinary approach to prevent potential medicolegal issues, contributions to emergency care education, and the assurance of safe patient care with high levels of patient and staff satisfaction [29].

Communication between emergency radiologists and emergency physicians, including immediate clarification of findings and collaborative decision-making through real-time discussions, can be conducted via telephone calls, text messages, emails, secure chat tools, internal messaging applications, and face-to-face communication [30]. Verbal communication is typically reserved for life-threatening critical findings due to the time-consuming and interruptive nature of other tasks [31].

Emergency physicians should provide clinical context, specific concerns, and relevant patient history to radiologists to prioritize cases and tailor interpretations. Conversely, emergency radiologists’ reports should include a clear summary of findings, highlighting critical results and suggestions for further evaluation or management [32]. Structured reporting templates are recommended to standardize reporting styles and provide key information in a readily accessible format [33]. Well-established lines of communication between emergency radiologists and physicians are essential for appropriate preliminary report turnaround times [30]. Mistakes and delays in communication between emergency radiologists and physicians can result in inappropriate emergency care management and pose medicolegal risks to both the radiologist and the institution [32]. Preparedness for acute emergency settings, including MCIs, necessitates an understanding of each other’s roles and responsibilities during regular interdisciplinary meetings and training sessions.

### Artificial intelligence in emergency radiology

The growing demand for imaging in emergency departments has placed substantial pressure on radiology services, leading to a non-negligible and increased workload for the emergency radiology department [34, 35]. This escalating burden often results in prolonged overall radiology services, including image acquisition, interpretation times, heightened cognitive fatigue, and a greater risk of diagnostic errors, particularly during high-volume periods and off-hours [36–38]. Optimizing operational and diagnostic workflows with precision in this high-stakes environment is essential to improve efficiency, reduce errors, and help achieve better patient outcomes.

**Fig. 5:**
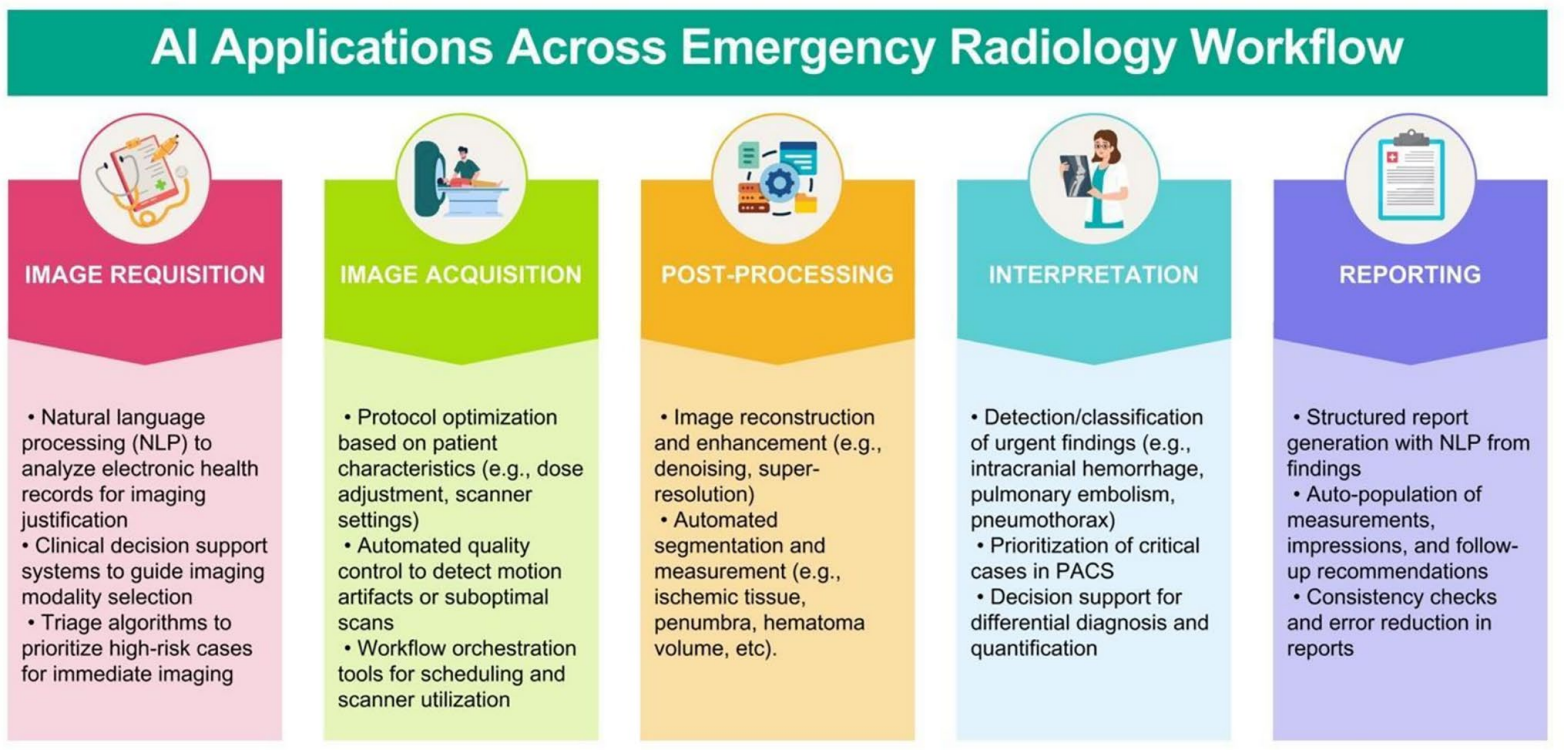
Potential artificial intelligence (AI) applications across emergency radiology workflow

With several potential interpretative and non-interpretative applications (Fig. 5), AI has emerged as a transformative tool and holds considerable potential to address longstanding challenges in emergency radiology by enhancing diagnostic accuracy, reducing delays, and supporting workflow optimization. Supporting this, a recent review of 882 FDA-reviewed AI products revealed that 154 tools (25%) had applications in emergency medicine, most of which were related to radiology [39].

In terms of the interpretative domain, AI algorithms can rapidly analyze imaging data, flag critical findings, and assist radiologists in prioritizing urgent cases, thereby reducing turnaround times and enabling timely interventions [40, 41]. Numerous studies have demonstrated AI’s ability to detect key pathologies with a level of performance comparable to experienced radiologists. Furthermore, AI tools have shown the potential to reduce missed diagnoses by physicians, particularly those early in their careers, by flagging overlooked abnormalities [42]. Common interpretative applications include intracranial hemorrhage, pulmonary embolism, and acute ischemic stroke [41, 43–49]. In some applications, AI tools provide quantitative assessments, such as ASPECTS scoring in acute stroke management, contributing to greater consistency and aiding decision-making [49].

Despite these promising potential applications along with popular fears and concerns, there is a growing consensus that AI will not replace radiologists in the near to medium term, but rather support them, especially by reducing error rates and interpretation times [50].

Beyond diagnostic tasks, AI offers potential value in non-interpretative domains that improve workflow efficiency. These include triage systems that prioritize critical studies for review [51, 52], protocol assistance based on clinical indications [53], patient positioning [54], and quality control tools that assess image adequacy before interpretation [55]. Moreover, AI can automate routine post-processing tasks, such as measurements and reconstructions, further alleviating the workload and streamlining clinical operations [56, 57].

Despite these advantages, the integration of AI into emergency radiology practice faces several challenges. Realizing this potential at scale will require careful attention to technical, clinical, financial, and ethical considerations [58]. A key barrier is clinician skepticism, driven by limited understanding of AI systems, concerns about diagnostic accountability, and the black box nature of many algorithms [59, 60]. Currently, most AI models are designed to address specific pathologies, and their suboptimal performance in rare or complex cases currently limits their role to diagnostic support rather than autonomous decision-making, a limitation that future foundation models may help overcome [61]. Financial considerations, including the cost of acquiring, implementing, and maintaining AI technologies, can also limit widespread adoption, particularly in resource-constrained settings [62–64]. Moreover, data-related limitations, such as imbalances in training datasets, variability in imaging protocols, and the need for secure data governance, pose significant risks to model generalizability and fairness [65–68]. Ethical and legal issues, including potential algorithmic bias, unclear lines of responsibility in case of error, and evolving regulatory frameworks, require proactive oversight and stakeholder engagement [69–72].

### Teleradiology considerations

Teleradiology involves the examination and reporting of radiological images from a different location than their original acquisition. Insufficient number of emergency radiologists available outside of normal business hours results in increased demand for teleradiology solutions, which may provide frontiers and cause drawbacks for radiology departments (Table 1). For many centers, the most important aspect of teleradiology is the evaluation of emergency radiological images. Recently, teleradiology companies have started offering emergency radiology software with AI and mobile apps to interpret emergency imaging studies. Reports indicate a higher use of teleradiology in emergency radiology within non-academic centers compared to university hospitals due to lack of residents and shortage of radiologists willing to work night shifts in rural areas [73].

**Table 1:** Emergency teleradiology considerations

Consideration		Potential advantages	Potential disadvantages
Remote reporting	Reporting of images obtained in the emergency radiology department outside of working hours	Uninterrupted service Quick reporting Reduced workload Reduced burnout in radiologists	Inaccurate or incomplete reporting Communication problems Seeing the radiologist as a commodity Remote access technical problems Financial problems Problems about the sharing of personal data
Rural hospitals	Shortage of radiologists	Uninterrupted service Quick reporting	Communication problems Remote access technical problems
Mass casualty incident	Radiology reporting support in extraordinary situations	Quick reporting with a large number of radiologists	Infrastructure and communication problems
Training/supervision	Remote support for radiology resident or inexperienced radiologists	Reduction in workload	Disruption in radiology resident training
Consultation	Consultation with specialized radiologists such as neuroradiologists	Increased diagnostic performance	Communication problems Extension of reporting period
Artificial intelligence	Preliminary evaluation of emergency radiological images with artificial intelligence software	Reduction in reporting time Increased diagnostic performance	Over-reliance Automation bias

In the realm of teleradiology, which is a large sector, ESR (European Society of Radiology) and ACR (American College of Radiology) have presented their opinions in the form of white papers at different times. Accordingly, it is stated that patient priority is the primary goal, with quality and safety being compatible with the hospital where the service is provided and complementary to the hospital where it is offered [74, 75]. In many countries, licensing is required for teleradiology services, and companies providing these services must comply with national laws, regulations, and guidelines often set by radiology associations. Institutions where teleradiology generates temporary reports should also consider implementing structured reports and following emergency radiology guidelines. For teleradiology, there are regulations in many countries regarding access to patients’ medical histories and the use of personal data. Previous studies have reported that the availability of past radiology images of patients seen by teleradiology can reduce costs and radiation doses by decreasing the need for new imaging [76, 77].

**Fig. 6:**
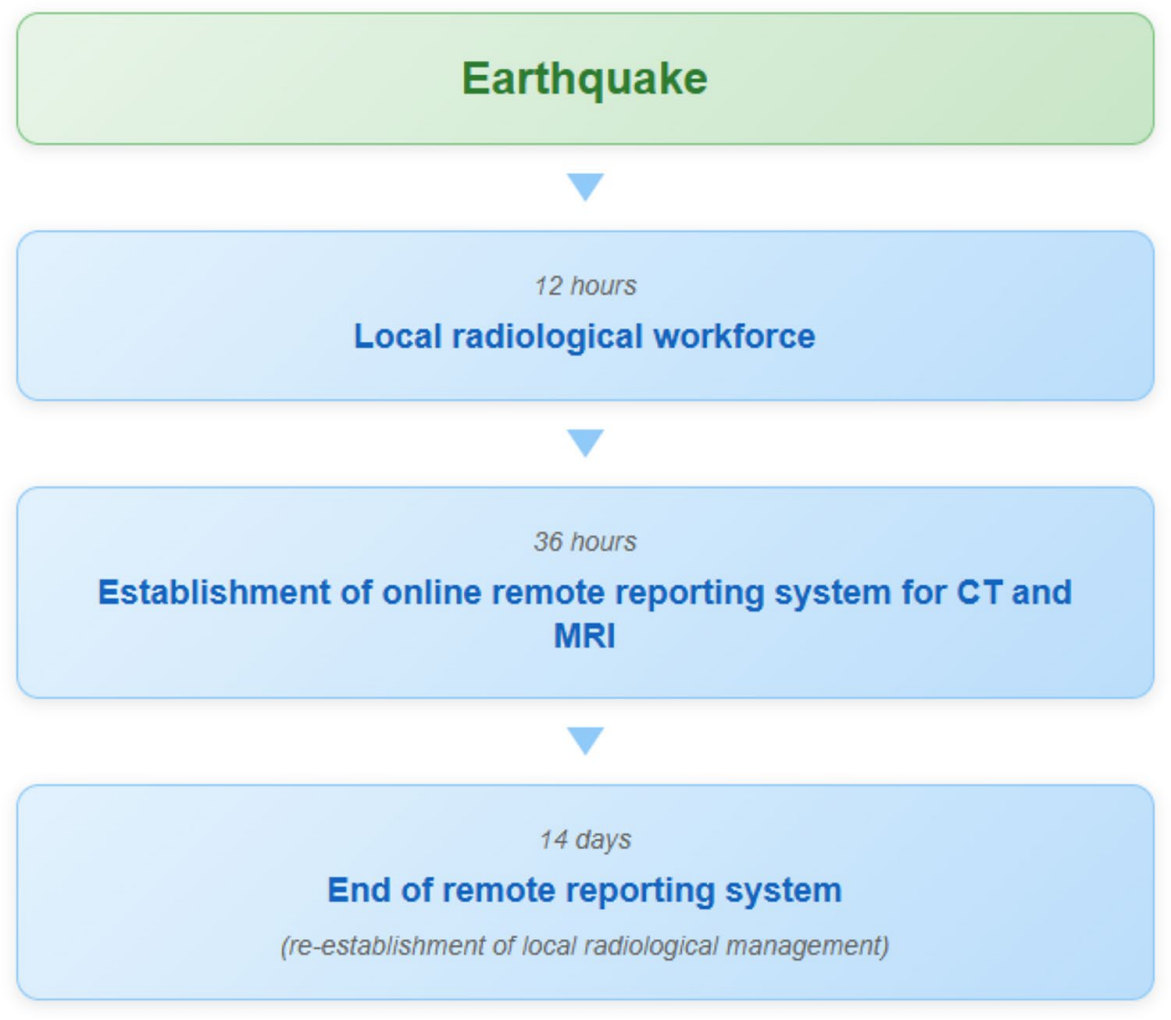
Timeline of the teleradiology system after twin earthquakes in Türkiye

With remote reading systems, high-quality image transfer, and PACS integration, remote radiologists can provide services of the same quality as those working on-site at the central hospital. Hybrid models that allow radiologists to work both on-site and remotely may improve the balance of work and increase staff satisfaction [78]. In addition, agreements with radiologists across different time zones for remote reporting may reduce the need for night shifts [10]. A pool of expert radiologists can provide 24/7 access for the interpretation of rare or complex cases [8]. The use of teleradiology in emergency radiology for disaster management greatly contributes, especially during earthquakes and wars. Earthquakes rank among the most devastating and erratic natural calamities. A magnitude of 7.7 foreshock transpired near the epicenter of the Kahramanmaraş earthquake at 04:17:35 (UTC + 03:00) on February 6, 2023. Subsequently, at 13:24:49 (UTC + 03:00), a magnitude of 7.6 earthquake transpired, with its epicenter located in Kahramanmaraş-Elbistan. The natural disaster ravaged 10 cities, and thousands of injured persons were transported to hospitals across Türkiye. In the initial month following the earthquake, reports indicated that 46,104 individuals had died and over 100,000 were injured due to the tremors originating in Kahramanmaraş [79]. While there was a shortage of regional radiologists following the earthquake that affected Türkiye and Syria on February 6, 2023, CT and MR images in emergency radiology were quickly evaluated and reported using the teleradiology method [80]. Despite attempts to engage the local workforce during the initial 12 h post-earthquake, the swift exceedance of capacity necessitated the activation of the teleradiology service within 36 h, facilitated by the proactive collaboration of the Ministry of Health and volunteer radiologists. Four days after the earthquakes, a teleradiology system was established with collaboration of 650 radiologists in Türkiye and abroad through which more than 20,000 CT and MRI examinations were reported within 48 h in the following days [80]. This service was efficiently sustained for 14 days before the application was discontinued with the re-establishment of local service supply (Fig. 6). During the COVID-19 global pandemic, the increase in imaging volume in emergency departments was matched by an increase in diagnostic interpretations by emergency radiologists working from home [81].

## Conclusion

Emergency radiology is a vital sub-discipline of radiology that caters to a vulnerable population requiring urgent medical attention, significantly contributing to the reduction of death and morbidity rates.

Radiologists engaged in emergency radiology operate under heightened pressures, mandated to deliver rapid and precise services around the clock, and have become essential members of the emergency team. Increased workload, dynamic and unpredictable, sometimes chaotic, noisy, and stressful nature of emergency imaging, as well as the need for flexible working hours enhanced with shifts, necessitate a well-organized emergency radiology unit.

The preparedness of emergency radiology departments for MCIs and their efficient collaboration with the emergency medical team mostly fall under the purview of department heads. Emergency radiologists are responsible for interpreting images, organizing emergency staff working hours, planning equipment, determining imaging protocols, establishing standardized reporting styles, tailoring imaging protocols, and communicating with emergency care physicians.

Despite its challenges, the use of AI in emergency radiology settings may enhance the speed and accuracy of diagnosis, as well as countless non-interpretative tasks. The efficient application of teleradiology decreases reporting workload in instances such as MCI and routine practice, supporting more timely diagnosis.

This review aims to provide readers with up-to-date overviews of the aforementioned topics, and all the co-authors are confident that it will be a good resource for all the stakeholders with an interest in the field.
